# Homozygous truncating *NEK10* mutation, associated with primary ciliary dyskinesia: a case report

**DOI:** 10.1186/s12890-020-1175-1

**Published:** 2020-05-15

**Authors:** Fuad Al Mutairi, Randa Alkhalaf, Abdullah Alkhorayyef, Fayhan Alroqi, Alyafee Yusra, Muhammad Umair, Fetaini Nouf, Amjad Khan, Alharbi Meshael, Aleidi Hamad, Alaujan Monira, Abdulaziz Asiri, Kheloud M. Alhamoudi, Majid Alfadhel

**Affiliations:** 1 Medical Genetics Division, Department of Pediatrics, King Abdullah specialized Children’s Hospital, King Abdulaziz Medical City, P. O Box 22490, Riyadh, 11426 Saudi Arabia; 2 Medical Genomics Research Department, King Abdullah International Medical Research Center (KAIMRC), King Saud Bin Abdulaziz University for Health Sciences, Ministry of National Guard Health Affairs (MNGH), Riyadh, Saudi Arabia; 3 Pulmonary Division, Department of Pediatrics, King Abdulaziz Medical City, Riyadh, Saudi Arabia; 4 Immunology Division, Department of Pediatrics, King Abdulaziz Medical City, Riyadh, Saudi Arabia

**Keywords:** NEK10, Immotile-cilia syndrome, Homozygous nonsense variant, Respiratory issues, Genetic testing, Primary Ciliary dyskinesia

## Abstract

**Background:**

Primary Ciliary Dyskinesia (PCD) is also known as immotile-cilia syndrome, an autosomal recessive disorder of ciliary function, leading to mucus retention in the respiratory system in childhood. Our knowledge in the pathophysiological aspect of this devastating disorder is increasing with the advancement of genetic and molecular testing.

**Case presentation:**

Here in, we report two siblings with a classical clinical and radiological presentation of PCD. Using whole exome sequencing we identified a homozygous truncating variant (c.3402 T > A); p.(Tyr1134*) in the *NEK10* gene. Western bolt analysis revealed a decrease in the expression of NEK10 protein in the patient cells.

**Conclusions:**

NEK10 plays a central role in the post-mitotic process of cilia assembly, regulating ciliary length and functions during physiological and pathological status. This study highlights the challenges of identifying disease-causing variants for a highly heterogeneous disorder and reports on the identification of a novel variant in *NEK10* which recently associated with PCD.

## Background

Cilia are hair-like organelles or structures that extend from the surface of nearly all mammalian cells. They are classified into three different classes such as primary cilia, which are immotile and expressed on many cells during development, nodal cilia found in the embryonic node and motile cilia, which are long thin protrusions extending up to 20 mm along the cell surfaces of respiratory epithelium, and falloppian tubes [1, 2]. PCD is also known as immotile-cilia syndrome. It is a rare genetic disorder, inherited in an autosomal recessive fashion that is characterized by progressive recurrent sino-pulmonary disease due to abnormal ciliary structure, function and movement leading to mucus retention and impaired mucociliary clearance in the respiratory system. In PCD, the motile cilia are mainly immotile, however stiff, uncoordinated, and/or ineffective ciliary beats have also been reported [3–5].

The prevalence of PCD estimated to be 1:10,000 to 1:20,000 live births, although this varies between different ethnic groups [6]. In the early course of the disease, lack of variability in the clinical presentation makes it difficult to diagnose and the disease is usually diagnosed after infancy or during early childhood. This causes a delay in the regular patient follow-up and adequate treatment [7]. The main PCD manifestations include recurrent infections of the upper and lower respiratory tracts that lead to chronic changes and ultimately progress to severe bronchiectasis at an older age [8]. Many genes have been associated with PCD as a result of modern next generation sequencing technologies, still the molecular diagnosis is possible for only ~ 70% of affected patients [2]. The links between specific genetic mutations and their association with transmission electron microscopy (TEM), immunofluorescence (IF), and video microscopic phenotype are well known, however there is no genotype-phenotype correlation for many new genes [9].

Recent findings suggest a key role of altered ciliogenesis or dysfunctional cilia as a cause of a wide range of genetic diseases [10]. NIMA-related kinases or NEKs are a group of less-characterized cell cycle kinase family. These members have key roles in mitosis; involved in ciliogenesis and some members play additional roles in ciliary function [11–13]. In a recent report, NEK10 deficiency was described as a novel human disease characterized by pathologically short motile cilia caused by impairment of the motile ciliary proteome which is responsible to promote ciliary length and mucociliary transport but which is dispensable for normal ciliary number, radial structure, and beat frequency [14].

Here in, we report an additional two siblings presenting recurrent chest infection, clinical and radiological findings consist of bronchiectasis with primary ciliary dyskinesia. Molecular analysis revealed a biallelic nonsense mutation in the *NEK10* gene located on chromosome 3p24.1.

## Case presentation

**Fig. 1:**
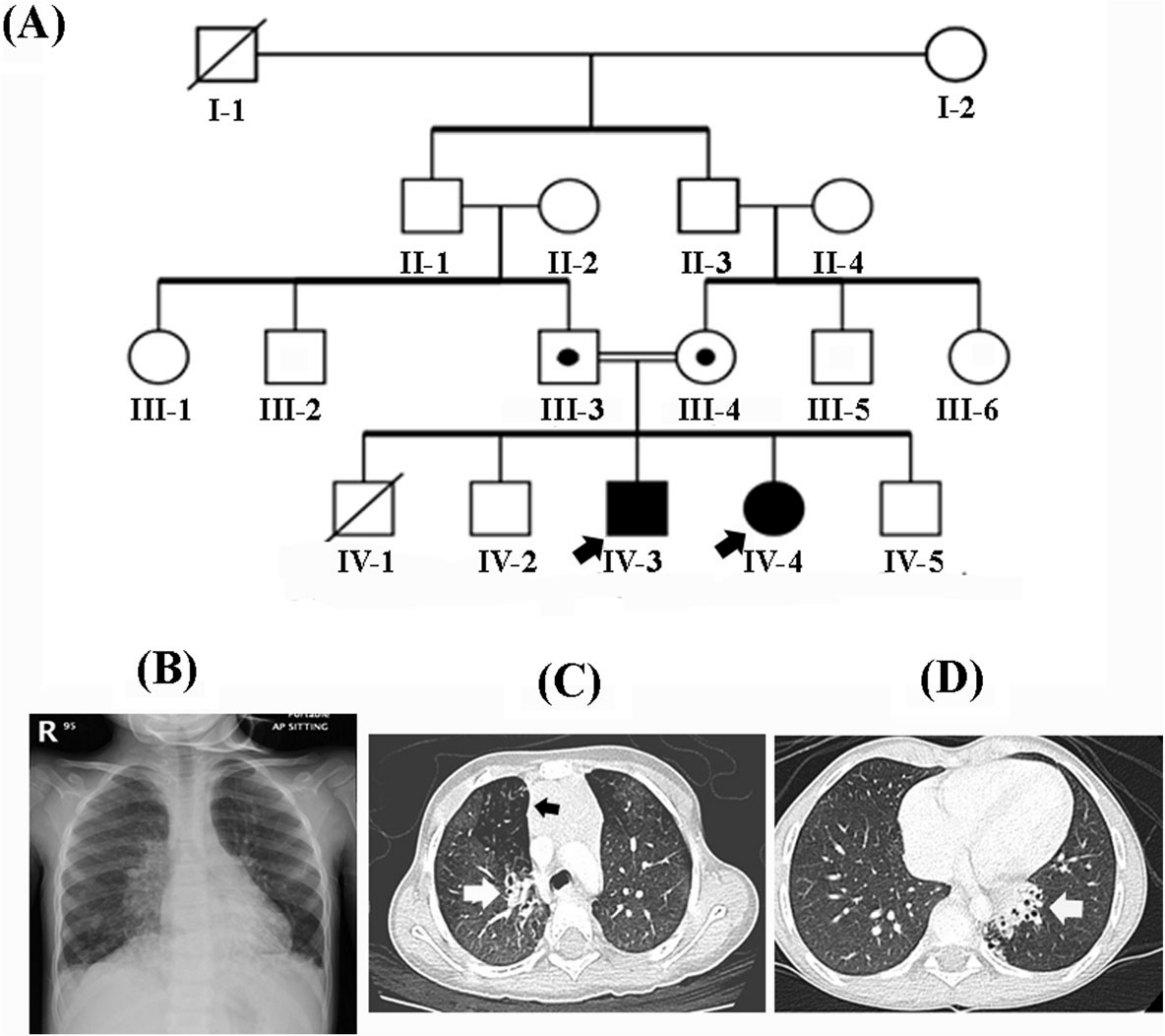
**a** Pedigree of the family showing consanguineous union and recessive inheritance pattern. **b** Chest X-ray of the affected individual (IV-4) revealed bilateral para-cardiac patchy infiltration with blunting of the left CP angle. **c**, **d** CT scan for affected individuals (IV-3) & (IV-4) showing mediastinal lymph nodes enlargement and bronchiectasis changes involving left lower lobe/ lingual, right upper lobe, and lateral segment of middle and medial segment of right lower lobes. White arrows depicting bronchiectasis (**c**, **d**), while the black arrow shows mosaic appearance (**c**)

Two siblings from a Saudi consanguineous family were presented to our practice. Their parents were first-degree consanguineous couple with two additional healthy children and previous history of terminated pregnancy at 5th months of gestation by intrauterine fetal death (Fig. 1a). Both affected siblings underwent a carful clinical evaluation by a pulmonologist, immunologist, and clinical geneticist.

The proband is a 5 years old girl (IV-4) born at full term by normal vaginal delivery. At 2 months of age, she developed recurrent presumed viral associated wheezing. She required hospitalization as she developed an increase in the severity of her respiratory episodes associated with hypoxia which need prolonged admission courses. She continued to have a chronic wet cough, recurrent otitis media, had multiple admissions for respiratory exacerbation. Once she was admitted to an intensive care unit, where she required non-invasive positive pressure ventilation and bronchodilator therapy and later discharged on oxygen at home. Developmentally, all her millstone domains were appropriate for her age. No other neurological or renal symptoms were observed. On examination, her weight was 13.5 kg (10th centile), height 98 cm (25th centile) and her head circumference 50 cm (between 50th–75th centile). Auscultation for her chest revealed an equal bilateral coarse breath sounds with diffuses crackles, while all other systemic examinations were unremarkable. A milder phenotype noticed in her elder 8 years old brother (IV-3) who did not require admission, therefore CT chest requested confirmed bronchiectasis.

The patient (IV-4) chest X-ray demonstrated bilateral para-cardiac patchy infiltration with blunting of the left CP angle. (Fig. 1b). CT chest for (IV-3, IV-4) showed bronchiectasis changes involving lower lobes, right middle and lingula with hilar and mediastinal lymph nodes enlargement (Fig. 1c,d). Upper GI study demonstrates mild gastroesophageal reflux, without evidence of pulmonary aspiration, or evidence of tracheoesophageal fistula (TEF). Sweat chloride test revealed 20 mmol/L (40 mmol/L), Total IgE 15.90 KU/L (5–22 KU/L). P-ANCA and C-ANCA were 2.30 Units and 2.39 Units respectively (< 20 negative). Lymphocyte subsets, immunoglobulins, specific antibody titers, oxidative burst test, and total complement activity (CH50) were all unremarkable.. Bronchoscopy showed normal airway anatomy with scattered thick whitish secretion bronchoalveolar lavage (BAL) taken and cultures were negative for bacterial, fungal and mycobacterium. Laparoscopic lung biopsy revealed histiocytic, lymphoplasmacytic infiltrate and lymphoid aggregates, no evidence of granuloma or malignancy was observed. Due to technical issues and limited resources, we could not perform ciliary EM, nasal nitric oxide (nNO), ciliary high-speed video microscopy (HSVM), ciliary beat pattern (CBP) and frequency (CBF).

The present family was subjected to Whole Exome Sequencing (WES) using standard methods [15]. Step-by-step filtering and validation of different homozygous and compound heterozygous variants revealed a nonsense variant (c.3402 T > A); p.(Tyr1134*) in the exon 37 of the *NEK10* gene (NM_152534.4; NP_955379.2). Using Sanger sequencing, the identified variant segregated perfectly with the disease phenotype within the family. The variant was present in the heterozygous state in the obligate carriers of the families. To exclude the non-pathogenic nature of the identified variant, it was screened within 2000 Saudi exomes, ExAC and gnomAD databases. The pathogenicity index was calculated using different online analysis tools [(MutationTaster: Disease causing, FATHMM-MKL: Damaging, Varsome: PM2, PP3, DANN: 0.9924)] and was predicted disease causing.

**Fig. 2:**
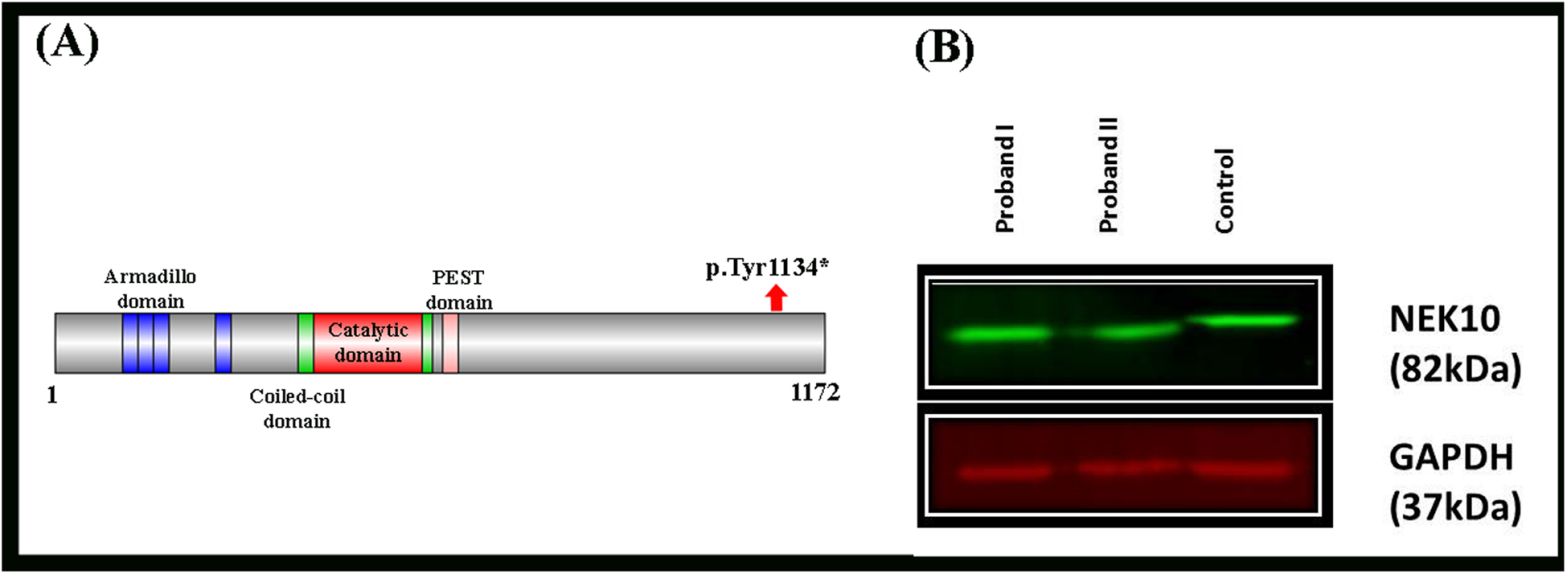
**a** NEK10 protein domains and position of the identified mutation. **b** Western blot analyses of a nonsense mutation in the *NEK10* gene [p.(Tyr1134*)]. Fibroblast cell lysates were subjected to Western blot analyses with anti-NEK10 and anti-GAPDH antibodies (loading control). NEK10 expression (82 kDa) was detected in all samples. A full-length protein size was found in the control sample, while a slightly reduced band size by ~ 4 kDa (pulled down bands) was observed in the affected sibling as compared to control

Furthermore, to prove the pathogenicity of this mutation, fibroblast cell lysates from both the affected individuals (IV-3, IV-4) were subjected to Western blot analyses with anti-NEK10 and anti-GAPDH antibodies (loading control) (Fig. 2b). NEK10 expression was detected in all the samples and a full-length protein size was found in the control sample, while a slightly reduced ~ 4 kDa was observed in the affected individual samples as compared to the control sample (Fig. 2b). Thus, the 39 amino acid small affected protein supporting the pathogenicity of the identified mutation [p.(Tyr1134*)].

## Discussion

Primary cilia have key roles in regulating signaling pathways that are initiated at the cell surface. Any pathogenesis or defect in the primary cilia function or structure underlie a wide range of inherited ciliopathies disorders [15]. During embryogenesis, the motile cilium structure is composed of nine peripheral doublet microtubules and two central single microtubules. They generate a whirling, rotational movement that directs the leftward flow of extracellular fluid and play a vital role in establishing left–right body orientation, and abnormalities can lead to laterality defects that include situs inversus [16].

Whole exome sequencing (WES) has been an extremely effective and quick method in the identification of disease causing variants in several complex genetic disorders. Here, we investigated single-family exhibiting classical features such as recurrent chest infection, and bronchiectasis. Using standard WES followed by Sanger sequencing, we elucidated a homozygous nonsense mutation (p.Tyr1134*) in the *NEK10* gene. The identified mutation was present in the C-terminal domain of the NEK10 protein. This mutation produced a premature stop codon at amino acid 1134, thus reducing the final protein size by 39 amino acids (total size 1174 amino acids). This mutation might affect the secondary structure of the NEK10 protein and might affect specific functions.

Mutations in the ciliary protein result in a number of ciliopathies, including retinal degeneration, polycystic kidney, liver and pancreatic diseases, abnormalities in neural tube closure and skeletal defects [17]. The importance of NIMA-related kinases (NEKs) in the cilia were first revealed through studies in the ciliated unicellular eukaryotes [18]. It has been proposed that the ability to coordinate the primary cilium with the cell cycle coevolved with the expansion of the NEKs family [19]. Experimental studies on mouse models have led to the identification of mutations in some of NEKs; which were important for post-mitotic process of cilia assembly, regulating the ciliary length and its proper function [20–24]. Human cells express eleven genes that encode NEK1 to NEK11, which contain an N-terminal catalytic domain having all the motifs that are typical serine/threonine kinase except NEK10, which have a centrally located kinase domain [25]. NEK10 is expressed significantly and uniformly along the axoneme of cilia, and has a key role in the G2/M checkpoint control, hence required for cilia assembly and biogenesis. While, the expression of a kinase-dead mutant of NEK10 (NEK10-KD) has significantly reduced the number of ciliated cells [24, 26].

The impact of NEK10 on cell growth in the ciliary compartment has revealed binary interaction between Pericentriolar matrix protein 1 (PCM1) and both cAMP-dependent protein kinases (PKA and NEK10). These interactions improved cilia dynamics via compartmentalized signaling networks, which in turn regulate proper cilia formation [27, 28]. Although the central role of.

NEK10 in the potentiation of mucociliary clearance was suggested, the mechanistic basis for this activity remains unclear because of difficulty to distinguish between direct phosphorylation effects and secondary protein abundance changes [14]. However pathogenic mutations affecting any component of this proteolytic machinery may alter the sensitivity of the cells to hormones and growth factors in both physiological and pathological conditions [24].

## Conclusion

In the present investigation, we report on two affected individuals having PCD, exhibiting poor weight gain and familial bronchiectasis. This is a confirmatory report, adding more evidence to recently published data suggesting the involvement of NEK10 loss of function mutation causing PCD in humans. This report further supports the candidacy of *NEK10* gene in the etiology of PCD and might facilitate in the identification of additional cases to further delinate the phenotype of this disorder. Our study also highlights the occurrence of clinical and genetic heterogeneity in such complex disorder and the importance of comprehensive clinical phenotyping to reveal the underlying molecular. Nevertheless, additional cases are required to framework proper genotype/phenotype correlation and design further specific therapeutic strategies.

## Data Availability

All data generated or analyzed during this study are included in this published article. Besides, any additional data/files may be obtained from the corresponding author.

## Abbreviations

PCD: Primary Ciliary Dyskinesia
NEKs: NIMA-related kinases
TEM: Transmission electron microscopy
IF: Immunofluorescence microscopy
PCR: Polymerase chain reaction
CT: Computed tomography
TEF: Tracheoesophageal Fistula
nNO: Nasal nitric oxide
BAL: Bronchoalveolar lavage
EM: Electron microspore
CBP: Ciliary beat pattern
CBF: Ciliary beat frequency
WES: Whole Exome Sequencing
PCM1: Pericentriolar matrix protein 1
